# Characteristics of humoral responses to the first coronavirus disease booster vaccine and breakthrough infection in central China: a multicentre, prospective, longitudinal cohort study

**DOI:** 10.3389/fimmu.2024.1446751

**Published:** 2025-01-07

**Authors:** Junhong Xu, Youhua Yuan, Guohua Chen, Bing Ma, Yin Long Zou, Baoya Wang, Wenjuan Yan, Qi Zhang, Qiong Ma, Xiaohuan Mao, Huiling Wang, Yi Li, Xiaohuan Zhang

**Affiliations:** ^1^ Department of Clinical Microbiology, Henan Provincial People’s Hospital, People’s Hospital of Zhengzhou University, and People’s Hospital of Henan University, Zhengzhou, China; ^2^ Department of Special Laboratory, Henan Provincial People’s Hospital, People’s Hospital of Zhengzhou University, and People’s Hospital of Henan University, Zhengzhou, China; ^3^ Department of Laboratory, Zhengzhou Municipal Chinese Medicine Hospital, Zhengzhou, China; ^4^ Department of Laboratory, Dengzhou Municipal Central Hospital, Dengzhou, China; ^5^ Department of PCR, Henan Provincial People’s Hospital, People’s Hospital of Zhengzhou University, and People’s Hospital of Henan University, Zhengzhou, China

**Keywords:** breakthrough infections, COVID-19, humoral immunity, kinetics, neutralising antibodies, booster immunization

## Abstract

**Introduction:**

The long-term immunogenicity, adverse effects, influencing factors, and protection from booster vaccines remain unclear. Specifically, little is known regarding the humoral immunity and breakthrough infections associated with COVID-19 booster immunization. Therefore, we evaluated the immunogenicity, reactogenicity, influencing factors, and protective effects of the first coronavirus disease booster vaccine 23 months before and after implementation of dynamic zero epidemic control measures among healthcare staff.

**Methods:**

We prospectively included 389 healthcare staff members in China with negative pre-enrolment severe acute respiratory syndrome coronavirus 2 test results. Neutralising serum antibodies were evaluated every two months till 23 months post-booster vaccination. Breakthrough infection was recorded or confirmed by SARS-CoV-2 specific PCR testing via throat swabs from participants before and after dynamic zero epidemic control measures.

**Results:**

At 15–30 days after vaccination, the mean concentration of the booster vaccine was 6.4 times above initial concentrations. Poorer antibody responses by booster vaccine correlated with male sex, longer post-booster duration, same-manufacturer vaccines, post-routine epidemic control measures implementation and intervals >210 days between primary and booster vaccinations. Higher breakthrough rates were associated with longer post-booster durations and post-routine epidemic control measures implementation but not associated with levels of neutralising antibodies after booster vaccination from participants. Adverse reactions were non-serious. These booster vaccine doses induced rapid, robust antibody responses, maintained for only 6–7 months.

**Discussion:**

Neutralizing antibodies induced by breakthrough infection with SARS-CoV-2 were weaker than those induced by the first COVID-19 booster vaccine, predicting that antibodies induced by SARS-CoV-2 may be very different from those of other known infectious pathogens.

## 1 Introduction

Coronavirus disease (COVID-19) is an emerging respiratory disease caused by severe acute respiratory syndrome coronavirus 2 (SARS-CoV-2) and has become a common infectious disease with worldwide spread (1, 2). Administration of a primary and booster vaccination is one of the most effective measures to control the spread of infectious diseases, including COVID-19 (3, 4). We previously showed that neutralising antibodies elicited by inactivated SARS-CoV-2 vaccines decline within 6–8 months of a primary two-dose inactivated vaccine program (5). Therefore, to control the epidemic, the Chinese Government provided the first booster vaccination dose freely to all citizens beginning in October 2021 (6), when strict dynamic zero-epidemic control measures were implemented against COVID-19 (7). This strict epidemic control measures referred when any residents tested positive for COVID-19 in a residential community, the positive residents were immediately sent to a centralised isolation hospital for free isolation, treatment, and observation. Other residents in the community were required to undergo throat swab nucleic acid testing and isolation at home for seven consecutive days so as to ensure none of positive resident live in this community. Coverage of the first dose of COVID-19 booster vaccine has reached 71.7% of the population requiring vaccination in July 2022 (8). Beginning on December 13,2022, routine epidemic control measures against COVID-19 were implemented (9). However, the neutralising antibody response after the first booster vaccination and its protective effect after the implementation of routine epidemic control measures remain unknown (10).

Currently, more than four types of COVID-19 vaccines have been approved for use globally (11, 12): mRNA, adenovirus, inactivated virus, and recombinant protein vaccines. In China, apart from not being able to produce mRNA vaccines, the other three vaccines have been approved and produced by eight different manufacturers, including Beijing Kexing Zhongwei, Beijing Biologics, Changchun Biologics, Beijing Kexing, Wuhan Biologics, Lanzhou Biologics, Tianjin CanSino, and Anhui Zhifei. So far, only a small number of Chinese have been vaccinated by booster adenovirus or recombinant protein vaccine, and most have been vaccinated by booster inactivated virus vaccine.

Booster vaccinations have been shown to be effective against other infectious diseases (3, 13); however, whether or not they are effective against emerging diseases such as COVID-19 is unclear. Additionally, previous studies have shown that the antibody level after primary vaccination of the COVID-19 vaccine is associated with age, sex, blood type, BMI (body mass index), occupation, interval of doses, the type of vaccination, and most importantly, the decreasing levels of antibody with time (5). Furthermore, studies from other countries have shown that different types of booster vaccines produce higher antibody titers among participants than the homologous type of booster vaccine (14) and last only five months; after this period from the booster vaccination, the number of breakthrough infections began to increase (15). However, in China, whether the antibody levels and maintained duration of the COVID-19 vaccine after the booster vaccine are related to these factors and the characteristics of SARS-CoV-2 breakthrough infections have not yet been reported since the first booster vaccine before and after the routine control measures.

This study aimed to assess the immunogenicity, reactogenicity, influencing factors, neutralizing antibody kinetics, and protective effects of the first booster vaccine dose during 23 months among medical staff.

## 2 Materials and methods

### 2.1 Ethics statement

This study was approved by the Ethics Committee of Henan Provincial People’s Hospital (approval number 20210051, approval date: 24 May 2021) and complied with the principles of the Declaration of Helsinki and Good Clinical Practice.

### 2.2 Study design and participants

This multicentre, prospective, longitudinal cohort study was performed at Henan Provincial People’s Hospital and Zhengzhou Municipal Chinese Medicine Hospital in Central China.

Healthy medical staff (18-80 years of age) members who received the first dose of the COVID-19 booster vaccine within 1 to 690 days between 20 October 2021 and 16 September 2023 were recruited. Participants with documented reverse transcription (RT)-PCR-confirmed COVID-19 or who had received any other vaccine, such as hepatitis B, were excluded. Symptoms or signs of clinically typical acute respiratory diseases such as a body temperature higher than 38°C, cough, signs consistent with COVID-19, or any contraindications to receiving the booster vaccine (such as allergies or pregnancy) within 24 hours before the target study vaccine dose were excluded (15).

All recruitment criteria were provided in crowded public places or announced on social networks such as Wechat groups. Interested candidates were invited to contact the researcher directly; at the same time, interviews by researchers were scheduled to explain these selection criteria. All participants provided written informed consent prior to enrolment.

When collecting the blood of a participant previously vaccinated with a booster dose, the study staff enquired and recorded whether the volunteer was or had been infected with SARS-CoV-2 after the booster vaccination. Simultaneously, a throat swab was collected for SARS-CoV-2 PCR testing; those with positive results were confirmed to have a breakthrough infection. Additionally, the related participant information was recorded in an investigation questionnaire and subsequently transferred to an electronic Excel form, including name, telephone contact, sex, age, body weight, and height. Detailed and accurate information on COVID-19 primary and booster vaccinations, including vaccine manufacturers, vaccination dates, and whether the vaccine was the adenovirus or recombinant protein vaccine type, could be obtained via the mini-program of the COVID-19 vaccination record on the Alipay platform. Currently, Chinese eight different manufacturers, including Beijing Kexing Zhongwei, Beijing Biologics, Changchun Biologics, Beijing Kexing, Wuhan Biologics, Lanzhou Biologics produced inactivated COVID-19 vaccine, Tianjin CanSino produced adenovirus recombinant live attenuated vaccine, and Anhui Zhifei produced recombinant protein vaccine. If a volunteer’s booster vaccine and the primary vaccine are both of the homologous type of vaccine, such as an inactivated vaccine, they are classified into the homologous group, and conversely, they are classified into the heterologous group. The ABO blood types of the volunteers were determined using identification reagents.

Hospitalized patients or other study participants from November 2022 to April 2024 were considered as having positive SARS-CoV-2 tests based on positive SARS-CoV-2 PCR results and infectious disease reports from the PCR department of Henan Provincial People’s Hospital; these results were eventually reported to the related administration section of the Chinese CDC.

### 2.3 Experimental procedures

For each booster, the vaccine was given as a single intramuscular injection with the same or different primary dose. All participants underwent clinical evaluation and blood samples were taken on the day of evaluation for neutralising antibodies. Significant signs and symptoms were measured for 1–30 days, and any solicited and unsolicited adverse events were reviewed, and medical records were updated. Two-millilitre blood samples were collected from each volunteer every 2 months, with the anticoagulant EDTA added into each tube to centrifuge with 3000g for 10 minutes, then,1 ml of plasma was drawn into 2 separate tubes, frozen in the -80°C refrigerator. The duration of neutralising antibodies for each participant was determined as the time interval between the booster vaccination date and the blood collection date. All participants (those with the heterologous type of vaccine and those with the booster dose of the homologous type of vaccine) remained at their local community health centre for at least 30 minutes following vaccination for the investigation and recording of any adverse events. When a serious adverse event occurred, participants contacted the researchers and rated the intensity of the adverse event on a severity scale as follows:1 = mild; 2 = moderate; 3 = severe; or 4 = life-threatening. Adverse event definitions and the list of solicited adverse events are categorized by previous description (16).

The neutralising antibody was detected by the commercial enzyme-linked immunosorbent assay (anti-SARS-CoV-2 S kit (Shanghai GeneoDx Biotechnology Co., LTD., Shanghai, China). The kit detects neutralising IgG antibodies only against the SARS-CoV-2 spike protein receptor binding domain not against nucleoprotein, which are available in a universal microplate reader (DNM-9602; Beijing Pulong Co., LTD., Beijing, China). A value greater than 6.5 IU/mL is regarded as positive. According to the manufacturer’s protocols and instructions, values greater than 100 IU/mL were considered to be 100 IU/mL. ABO blood group was determined via the test tube method according to the reagent’s instructions (Chengdu United Co., LTD., Chengdu, China).

Throat swab samples were collected for SARS-CoV-2 analysis using RT-PCR (Shanghai Zhijiang Biotechnology Ltd.). Cycle threshold values of ≤44 on RT-PCR were counted as positive.

### 2.4 Statistical analysis

A multivariable linear regression model was used to analyse factors influencing the concentration of neutralising antibodies, with one of the classification variances used as a reference to calculate the B-value (17). Due to missing data for some neutralising antibodies, we used a mixed linear model that could handle the unequal number of repeated observations of individuals with randomly missing data. To analyse the change of neutralising antibodies over time, we used a mixed linear model with the continuous log2 conversion concentration of neutralising antibodies from day 1 to 690 as the dependent variable. Age, sex, BMI, vaccination method, duration since the booster dose, interval between primary and booster dose, with or without breakthrough of SARS-CoV-2 infection, epidemic control measures, and ABO blood group were independent variables in the model. Additionally, we used multivariate binary logistic regression analysis to get the risk factors associated with breakthrough infection with SARS-CoV-2. In this analysis, the outcome variable was SARS-CoV-2 infection with 1–690 days post-booster vaccination, while other factors, including blood type, BMI, sex, age, vaccination mode, log2-transformed concentration of neutralising antibodies, duration post-booster vaccination, the interval between primary and booster vaccination, and epidemic control measures were used as independent variables. To investigate any correlation between neutralising antibody concentration and breakthrough infection rate over time after booster vaccination, we used the trend chi-square test and linear correlation analysis. Additionally, survival curve analysis was used to compare whether there was a difference in breakout rates between the two modes of booster vaccination (heterologous and homologous types). Moreover, chi-square test was used to compare the difference of adverse effects between individuals vaccinated with booster vaccines from the homologous and heterologous types as the primary vaccine.

Based on methods described in a previous study (18), we divided participants into three BMI groups: <18.5, 18.5–23.9, and>23.9 kg/cm2. Additionally, based on the Chinese COVID-19 booster vaccination procedure (6–8 months), we divided the interval between primary and booster doses into two groups:180–210 days and >210 days. We divided the participants into three groups by age: 18–30, 31–50, and >50 years (5, 18). We divided the duration after booster vaccination into 10 groups: 1–14, 15–30, 30–90, 91–150, 151–210, 211–300,301–365,366–420, 421–480, and 481–690 days. Additionally, other variables, including sex, vaccination type, presence or absence of SARS-CoV-2 breakthrough infection, epidemic measures, and blood type, were divided into different categories based on their natural classifications. Mixed model distribution curves of log2-transformed neutralising antibodies responses with duration, adjusted for age, sex, BMI, ABO blood type, vaccination mode, the interval between primary doses and booster doses of vaccination, SARS-CoV-2 breakthrough infection, epidemic measures, and time elapsed since booster dose were plotted using Prism 8.0 (GraphPad Software Inc., La Jolla, CA). We only included variables that showed significant associations with neutralising antibodies in the mixed model, even if potential confounders were controlled by the statistical analysis.

The results of the adverse reaction analysis were expressed as a percentage of participants and included all participants who received their first dose of booster vaccine and experienced local or systemic adverse events for 30 consecutive days following vaccination.

Sample size calculation for a log2-transformed neutralizing concentration was done to assess the humoral immune response against SARS-CoV-2 spike protein during 1-690 days after the first booster dose of in participants that received a homologous booster type, as compared with heterologous type. A sample size of 360 participants (n=240 in the homologous group) was required to identify a 15% increase in antibody concentration between two different vaccination group during 1-690 days, assuming a coefficient of variation equal to 1·2 or 1·0 and similar between groups, at least 80% power and a two-sided 5% significance level. The sample size was increased by 15% due to possible loss of visit.

An independent data monitoring committee composed of independent scientists not involved in this study regularly reviewed the data for safety.

All analyses, including linear mixed models, logistic regression, and adverse effects analyses were conducted using SPSS (version 25.0; IBM Corporation, Armonk, NY), and figures were plotted using Prism 8.0 (GraphPad, Inc.).

## 3 Results

### 3.1 Basic characteristics of study participants

**Figure 1:**
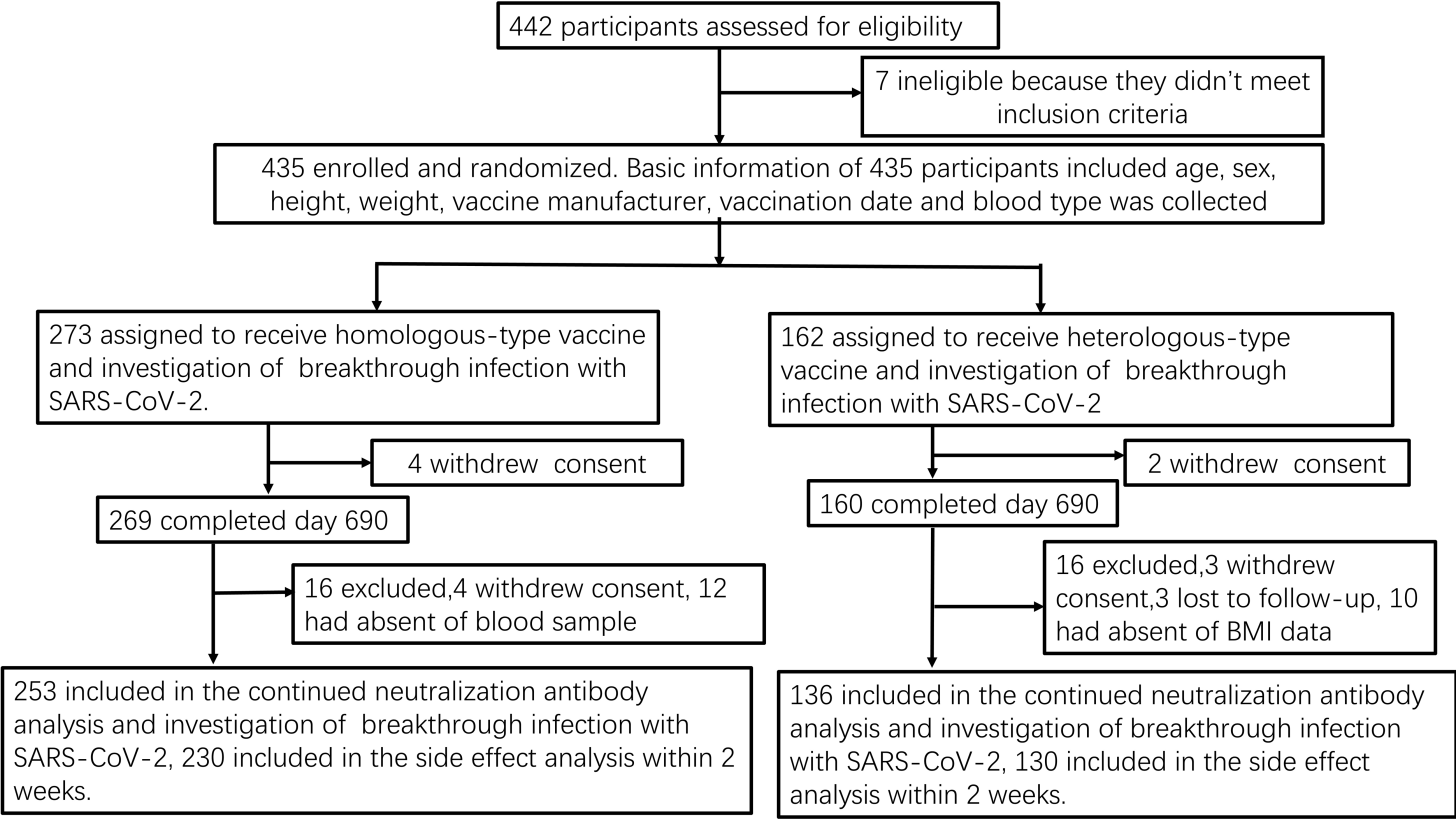
Study flow-chart Prospective cohort of Chinese individuals who received the booster vaccine against COVID-19 and underwent serological assays. Following vaccination, the participating medical staff members of Henan Provincial People’s Hospital and Zhengzhou Municipal Traditional Medicine Hospital in Central China were followed up at two-month intervals for 23 months between October 19, 2021, and September 30, 2023. BMI, body mass index; COVID-19, coronavirus disease; SARS-CoV-2, severe acute respiratory syndrome coronavirus 2.

We collected 791 serum samples from 389 medical staff members (**Figure 1**). Owing to missing data, we excluded 45 candidates from the regression and mixed model analyses:13 withdrew consent, seven did not meet the inclusion criteria, three missed a follow-up visit, 10 did not answer the body mass index (BMI) question, and 12 were not willing to provide blood samples (**Figure 1**). The concentration kinetics of neutralising antibodies were detected for all study participants at least once during the 690-day timeframe and a maximum of six times for 132 participants (33.9%). Before 13 December 2022, when the dynamic zero COVID-19 epidemic policy was implemented, 447 serum samples were collected from 113 participants, while 344 serum samples from 276 participants were collected after this date when the COVID-19 epidemic prevention and control measures were replaced with routine measures. Among 791 serum samples from 389 medical staff, 306 samples were confirmed to be SARS-CoV-2-positive by polymerase chain reaction (PCR) on the throat or memory information. The average breakthrough infection rate was 38.7% (306/791) during the study period (20 October 2021 to 16 September 2023). The cumulative SARS-CoV-2 infection rate for 276 samples collected from 13 December 2022 to 16 September 2023 was 86.2% (238/276). In contrast, before December 13, 2022, the cumulative infection rate was <8.1% (36/447). Baseline characteristics, including vaccination groups, age, breakthrough SARS-CoV-2 infection, and epidemic control measures, are indicated in **Table 1**.

**Table 1:** Baseline characteristics of study participants (N = 389).

Factor	Heterologous type (n = 136)	Homologous type (n = 253)	*p*	Overall
Sex				
Men	30	36	0.05	66
Women	106	217		323
Age (years), M (P25, P75) ^*^	25 (21, 44)	36 (26, 50)	0.001	33 (22, 49)
Age group (years)				
18–30	94	121	0.001	215
31–50	13	73		86
>50	29	59		88
Blood type				
A	33	64	0.833	97
B	38	62		100
O	51	104		155
AB	14	23		37
BMI (kg/m^2^)				
<18.5	11	16	0.196	27
18.5–23.9	96	162		258
>23.9	29	75		104
SARS-CoV-2 breakthrough infection				
Yes	95	142	0.008	237
No	41	111		152
Epidemic control measures against COVID-19				
Dynamic zero measures before 13 December 2022	25	88	0.001	113
Routine control measures after 13 December 2022	111	165		276

^*^M (P25, P75): median (interquartile range). BMI, body mass index; COVID-19, coronavirus disease; SARS-CoV-2, severe acute respiratory syndrome coronavirus 2.

### 3.2 Factors influencing neutralising antibody production after booster vaccination

**Figure 2:**
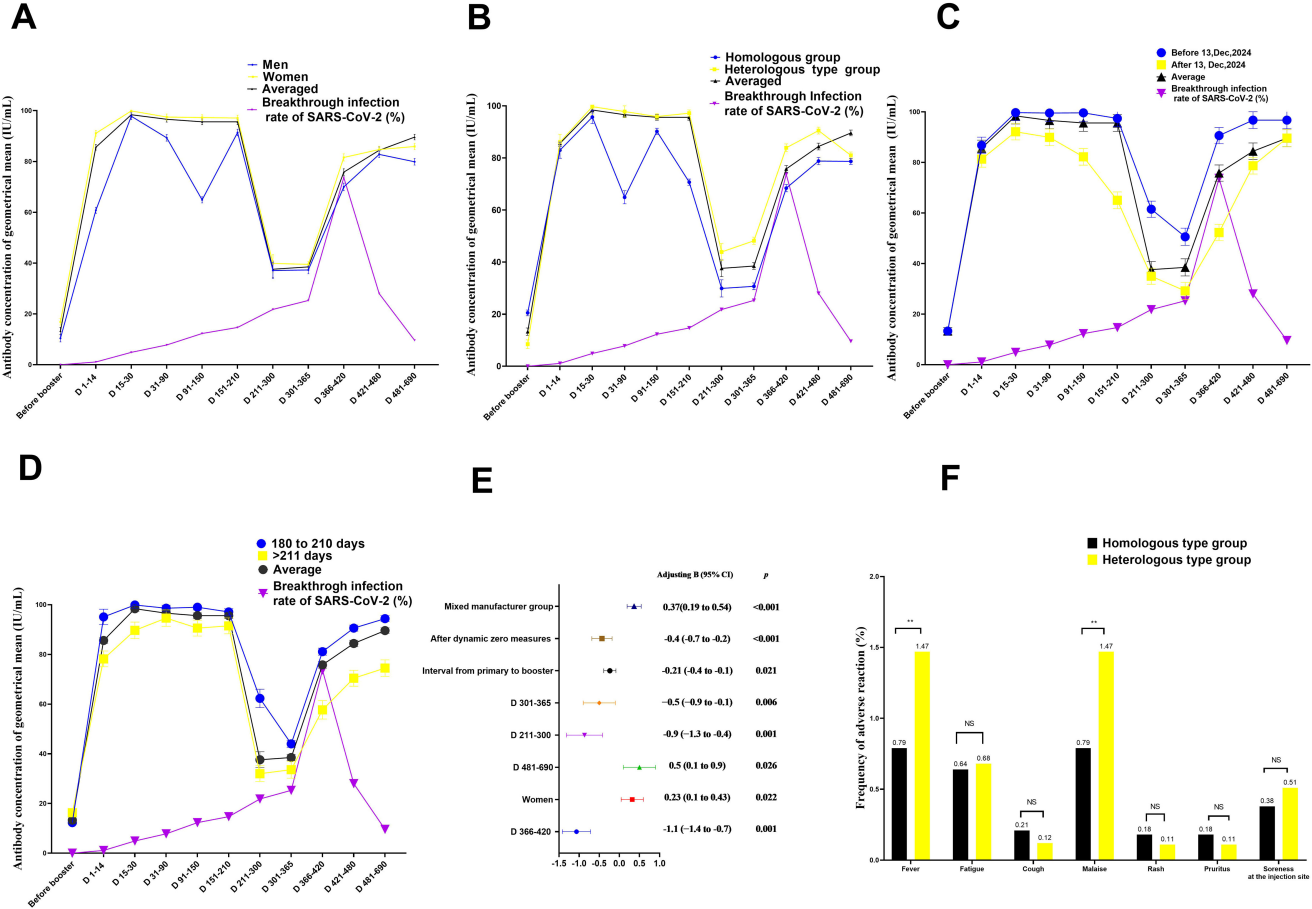
Quantitation of antibodies on days 1–690 following administration of the Chinese booster vaccine **(A–D)** Kinetics of neutralising antibodies according to **(A)** sex, **(B)** vaccination type, **(C)** control measures against COVID-19, and **(D)** interval between primary and booster vaccinations, **(E)** Factors influencing neutralising antibody production after booster vaccination in medical staff **(F)** comparison of adverse effects between individuals vaccinated with booster vaccines from the same and different types as the primary vaccine. Data are presented as mean (95% confidence interval [CI]) from the linear mixed-effects model adjusted for vaccine type, sex, blood type, age, interval between primary and booster vaccinations, COVID-19 control measures, and BMI. The log_2_-transformed levels of neutralising antibodies were used as independent variables. Chi-square test was used to compare the difference of adverse effects between individuals vaccinated with booster vaccines from the same and different types as the primary vaccine. BMI, body mass index; LSMD, least-squares mean difference; COVID-19, coronavirus disease; SARS-CoV-2, severe acute respiratory syndrome coronavirus 2.

Linear mixed model regression analysis showed that sex, interval between primary and booster vaccinations, COVID-19 epidemic control measures, vaccination duration booster, and vaccination type were significantly associated with neutralising antibody level (**Table 2**, **Appendix Tables 1**–**9**, and **Figures 2A–E**).

**Table 2:** Factors associated with neutralising antibody concentration after Chinese COVID-19 booster vaccine administration.

Factors	*n* (%)	B (95% CI)	*p*
Sex			
Men	66 (24.5)	Reference	
Women	323 (75.5)	0.23 (0.1 to 0.43)	0.022
Age (years)			
18–30	215 (55.3)	Reference	
31–50	86 (22.1)	0.08 (-0.63 to 0.06)	0.978
>50	88 (22.6)	0.18(-0.03 to 0.37)	0.074
Blood type			
A	97 (24.9)	Reference	
B	100 (25.7)	0.06 (-0.2 to 0.3)	0.504
O	155 (39.8)	-0.05 (-0.3 to 0.2)	0.427
AB	37 (9.5)	0.04 (-0.2 to 0.3)	0.272
Booster vaccination type			
Homologous type	253 (73.1)	Reference	
Heterologous type	136 (26.9)	0.37(0.19 to 0.54)	<0.001
BMI (kg/m^2^)			
<18.5	27 (6.9)	Reference	
18.5–23.9	258 (66.3)	-0.14 (-1.1 to 0.85)	0.053
>23.9	104 (26.7)	-0.05 (-1.1 to 0.9)	0.525
Duration since booster vaccination (Mean days)			
1–14	31 (7.9)	Reference	
15–30	33 (8.5)	0.2 (-0.04 to 0.4)	0.997
31–90	40 (10.3)	0.2 (-0.16 to 0.5)	0.287
91–150	28 (7.2)	0.1 (-0.1 to 0.4)	0.850
151-210	17 (4.4)	0.4 (-0.3 to 1.2)	0.257
211–300	25 (6.4)	-0.9(-1.3 to -0.4)	0.001
301–365	61 (15.7)	−0.5(-0.9 to -0.1)	0.006
366–420	63 (16.2)	-1.1(-1.4 to -0.7)	0.001
421–480	66 (16.9)	0.2(-0.2 to 0.4)	0.585
481–690	53 (13.6)	0.5 (0.1 to 0.9)	0.026
Interval between primary and booster vaccinations (Mean days)			
180–210	185 (47.6)	Reference	0.021
>210	204 (52.4)	-0.21 (-0.4 to -0.1)	
SARS-CoV-2 breakthrough infection			
Yes	237 (60.9)	Reference	0.378
No	152 (39.1)	-0.12 (-0.28 to 0.1)	
Epidemic control measures against COVID-19			
Dynamic zero policy before 13 December 2022	113 (29.0)	Reference	<0.001
Routine control measures after 13 December 2022	276 (71.0)	-0.4 (-0.7 to -0.2)	

BMI, body mass index; CI, confidence interval; COVID-19, coronavirus disease; SARS-CoV-2, severe acute respiratory syndrome coronavirus 2.

Neutralising antibody levels were 32.4 IU/mL higher in women than in men after adjusting for age, booster vaccine type, blood group, duration of primary and booster vaccination intervals, duration of vaccination, COVID-19 outbreak control measures, SARS-CoV-2 breakthrough infection, and BMI (B = 0.23; 95% confidence interval [CI], 0.11–0.43; P = 0.022; least square mean difference = 32.4; 95% CI, 22.3–45.1, P= 0.001) (**Figure 2A**, **Table 2**; **Appendix Table 5**). In addition, at days 31–90, the geometric mean concentration (GMC) of neutralising antibodies in the mixed vaccine group was 97.8 IU/mL, significantly higher than that in the same vaccine group (64.9 IU/mL) (P<0.001; **Figure 2B**, **Table 2**; **Appendix Table 4**). From days 211 to 300, the GMC of neutralising antibodies in both groups declined rapidly. After 210 days of booster vaccination, the GMC of neutralising antibody in the homologous vaccination group decreased to 29.9 IU/mL, which was significantly lower than that in the mixed vaccination group (43.9 IU/mL; P<0.001; **Appendix Table 4**; **Figure 2B**). In addition, we determined that the duration after booster vaccination was a major factor in the decline or rise of neutralising antibodies to booster vaccines; neutralising antibody GMC increased by 6.4 times; from 13.3 IU/mL before vaccination to 98.4 IU/mL 15 to 30 days after vaccination. GMC for all participants decreased from the highest level of 98.4 IU/mL at 15–30 days to the lowest level of 37.6 IU/mL at 210–300 days (mean decline:68% or 2.6-fold), indicating an average monthly decline of 8.7% (**Figure 2B**).

However, after the change in epidemic containment measures on 13 December 2022, the GMC of neutralising antibodies increased slightly again on days 301–366, from the lowest level to 86.9 IU/mL on days 481–690 (**Figures 2A–E**; **Appendix Tables 1**-**10**). This trend is consistent with research findings that the COVID-19 prevention and control measures was a major affect factor associated with neutralising antibodies. After strict control measures were removed, individuals produced fewer neutralising antibodies than before that date (B= -0.4; 95% CI, -0.7 to 0.2; P < 0.001; **Table 2**, **Figure 2C**). In addition, neutralising antibody levels were lower in patients with an interval of >210 days between primary vaccination and booster vaccination than in patients with an interval of 180–210 days (B=-021; 95% CI, -0.4 to -0.21; P= 0.021; **Figure 2D**, **Table 2**; **Appendix Table 8**). Neutralising antibody levels were not associated with SARS-CoV-2 breakthrough infection (B= -0.12; 95% CI, -0.28 to 0.10; P= 0.378; **Table 2**).

### 3.3 Characteristics of reactogenicity after the first booster vaccination

Adverse effect analysis was based on the solicited adverse events in 253 and 136 homologous- and heterologous-type vaccination groups, respectively, by 30 days after the first booster vaccination. In both groups, most adverse events were mild (n = 16, 76.2%) or moderate (n = 5, 23.8%), and self-limiting. The most common adverse effects were fatigue (n = 7), followed by fever (n = 4), pain at the injection site (n = 4), malaise (n = 4), rash (n = 2), and pruritus (n = 2). The incidence of fever and malaise in the heterologous-type vaccination group was slightly but significantly higher (2/136, 1.47%) (P<0.01) than that in the homorologous-type vaccination group (2/253, 0.79%; **Figure 2E**).

### 3.4 Factors influencing SARS-CoV-2 breakthrough infection after booster vaccination

**Figure 3:**
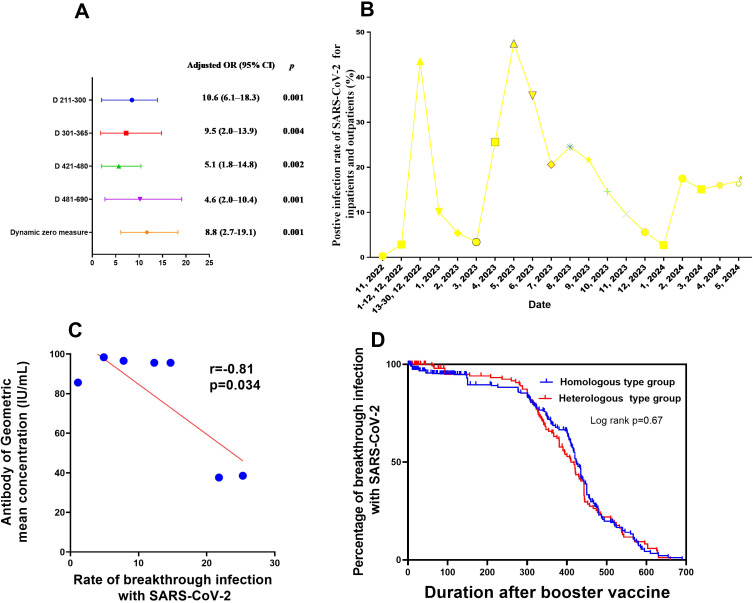
Factors influencing neutralising antibody production after booster vaccination, as well as SARS-CoV-2 breakthrough infection **(A)** Factors associated with breakthrough SARS-CoV-2 infection after booster vaccination in medical staff. **(B)** Rate of PCR-positivity for SARS-CoV-2 for inpatients and outpatients from Nov 2022 to Apr 2024. **(C)** Correlation between neutralising antibody production and breakthrough SARS-CoV-2 infection after booster vaccination in medical staff. **(D)** Survival curves of breakthrough infection between two modes of booster vaccination (mix and homologous types). CI, confidence interval; PCR, polymerase chain reaction; OR, odds ratio; SARS-CoV-2, severe acute respiratory syndrome coronavirus 2.

The results of the univariate and multivariate analyses to identify influencing factors associated with SARS-CoV-2 breakthrough infection after the first booster vaccination are shown in **Table 3** and **Figure 3A**. The following factors were found to be associated with post-booster vaccination SARS-CoV-2 breakthrough infection after univariate analysis: age, blood type, booster vaccination type, BMI, duration after booster vaccination, the interval between primary and booster vaccinations, COVID-19 epidemic control measures, and neutralising antibody concentration. Independent risk factors for SARS-CoV-2 breakthrough infection post-booster vaccination included COVID-19 epidemic control measures and duration after booster vaccination after multivariate analysis.

**Table 3:** Factors associated with breakthrough infection of SARS-CoV-2 after administration of the Chinese COVID-19 booster vaccine.

Factors	Infected person times (n =306) %	Uninfected person times (n = 485) %	Univariate analysis		Multivariate analysis	
			OR (95% CI)	p	OR (95% CI)	P
Sex						
Men	82 (26.8)	138 (28.5)	Reference	0.613	^*^	
Women	224 (73.2)	347 (71.5)	1.1 (0.8–1.3)		^*^	–
Age (years)						
18–30	124 (40.5)	132 (27.2)	Reference	<0.001	Reference	
31–50	92 (30.1)	86 (17.7)	0.88 (0.6-1.3)		1.1(0.5–2.1)	0.847
>50	90 (29.4)	267 (55.1)	2.8 (2.0-3.9)		0.9(0.5–1.8)	0.865
Blood type						
A	79 (20.83)	168 (29.17)	Reference	<0.001	Reference	
B	61 (31.94)	75 (36.11)	1.7 (1.1–2.7)		0.625(0.3–1.3)	0.191
O	115 (37.50)	100 (40.28)	2.4 (1.7–3.6)		0.959(0.5–1.8)	0.898
AB	51 (11.11)	142 (10.42)	0.7 (0.5–1.2)		0.674(0.3–1.4)	0.275
Booster vaccination type						
Homologous type	203 (66.3)	407 (83.9)	Reference	0.001	Reference	
Heterologous type	103 (33.7)	78 (16.1)	0.6 (0.5-0.8)		1.3(0.7–2.3)	0.351
BMI (kg/m^2^)						
<18.5	21 (6.9)	6 (1.3)	Reference	0.001	Reference	
18.5–23.9	173 (56.5)	328 (67.6)	6.6 (2.6–16.7)		0.9(0.3–2.8)	0.896
>23.9	112 (36.6)	151 (31.1)	4.7 (1.8-2.1)		1.5(0.5–4.9)	0.522
Duration since booster vaccination (Mean days)						
1–14	15 (4.9)	84 (17.3)	Reference		Reference	
15–30	4 (1.3)	77 (15.9)	0.29 (0.1–0.9)	<0.001	0.3(0.1–1.3)	0.107
31–90	10 (3.3)	119 (24.5)	0.5 (0.2–1.1)		0.5(0.2–1.2)	0.114
91–150	19 (6.2)	136 (28.0)	0.8 (0.4–1.6)		0.8(0.4–1.8)	0.604
151–210	3 (0.98)	4 (0.82)	4.2 (0.9–20.7)		1.5(0.2–10.3)	0.708
211–300	22 (7.2)	3 (0.62)	41.1 (10.9–54.6)		9.5(2.0–13.9)	0.004
301–365	58 (18.9)	10 (2.1)	32.5 (13.6–77.3)		5.1(1.8–14.8)	0.002
366–420	56 (18.3)	19 (3.9)	16.5 (7.7–35.2)		2.5 (0.97–5.3)	0.059
421-480	72 (23.5)	28 (5.8)	14.4 (7.1–29.0)		4.6(2.0–10.4)	<0.001
481-690	47 (15.4)	5 (1.0)	52.6 (18.0–54.0)		8.8(2.7–19.1)	<0.001
Interval between primary and booster vaccinations (Mean days)						
180-210	170 (55.6)	371 (76.5)	Reference	<0.001	Reference	
>210	136 (44.4)	114 (23.5)	1.4(1.2–1.5)		1.1(0.6–1.8)	0.844
Epidemic control measures for COVID-19						
Dynamic zero policy before 13 December 2022	36 (11.8)	411 (84.7)	Reference	<0.001	Reference	<0.001
Routine control measures after 13 December 2022	270 (88.2)	74 (15.3)	7.2(5.3–9.8)		10.6(6.1–18.3)	
Neutralising antibody concentration (IU/mL) Mean± SD	58.1 ± 2.8	87.1 ± 1.6	–	<0.001	0.9(0.7–1.1)	0.387

“*”: Multivariate analysis was not included, “-”: not available.

BMI, body mass index; COVID-19, coronavirus disease; CI, confidence interval; OR, odds ratio; SARS-CoV-2, severe acute respiratory syndrome coronavirus 2; SD, standard deviation.

The risk of post-booster vaccination breakthrough infection was at its highest at 7 months after booster vaccination (9.5 times higher than that at 15 days post-booster vaccination). Additionally, the SARS-CoV-2 breakthrough infection risk after implement of routine epidemic prevention and control measures was 10.6-fold higher than that of duration when dynamic zero policy was strictly implemented (**Table 3**, **Figure 3**). This tendency was consistent with the results of nucleic acid testing for hospitalized patients and other populations in Henan Provincial People’s Hospital from November 2022 to April 2024 (**Figure 3C**; **Appendix Table 10**). No link was found between SARS-CoV-2 breakthrough infection and GMC neutralising antibodies (adjusted odds ratio,0.9; 95%CI, 0.7–1.1, P=0.387; **Table 3**). However, within 6 months post-booster vaccination, the SARS-CoV-2 breakthrough infection rate increased with the decrease in GMC (linear correlation x2 = 318.7, P<0.001; r=-0.81, P=0.034, respectively; **Figures 2A–D**, **Figure 3C**). No difference was found in the rate of breakthrough infection among participants between the two modes of booster vaccination (heterologous and homologous types) (**Figure 3D**).

As shown in **Figure 3B** and **Appendix Table 10**, before 13 December 2022, when dynamic zero measures were implemented, the proportion of the entire population infected with SARS-CoV-2 was below 0.27%. Conversely, after 13 December 2022, with the release of epidemic measures, the SARS-CoV-2 infection rate among the overall population increased rapidly to 43.57%. After 6 months and up to May 2023, the number of hospitalized patients with SARS-CoV-2 further increased to 47.5%. However, during the subsequent 6 months up to November 2023, no outbreak of SARS-CoV-2 infections occurred, and the infection rate was maintained at about 20% among hospitalized patients as of April 2024. This implies that the neutralising antibody levels in the entire population may have been elevated to a level that protects against COVID-19 outbreaks, which is consistent with our finding that the neutralising antibody levels in medical staff or the general populations only maintained for 6–7 months after booster vaccination (**Figures 2A–D**).

## 4 Discussion

To the best of our knowledge, no previous study has reported on the induction of humoral responses in medical staff after the first dose of the Chinese COVID-19 booster vaccine before and after the implementation of dynamic zero measures. The findings of this study indicate an association between booster vaccination and an acceptable adverse effect spectrum during the 690 days following vaccination of heterologous type and homologous type. Additionally, this study identified that change of COVID-19 epidemic control measures and duration after booster vaccination were two influence factors associated with SARS-CoV-2 breakthrough infection within 690 days after booster vaccination in medical staff. To date, the lifting of dynamic zero control measures and a longer interval between primary and booster vaccination that reduced humoral immunogenicity, which led to a higher breakthrough infection rate, has not previously been reported by studies on COVID-19 booster vaccines.

We also found that after the lifting of COVID-19 epidemic control measures, neutralising antibody production in medical staff that had received the booster vaccine was lower than that before this time point, which is contrary to the traditional understanding of infectious diseases. The traditional theory posits that neutralising antibody production increases with a rising incidence of infectious diseases. (19) As we know, smallpox, measles, chickenpox, these infectious diseases are life-long antibody immunity, and the vaccine against these infectious diseases can only provide a maximum of 7 years of antibody protection, requiring regular booster vaccination (20–22). This finding may be explained by the fact that the immune effect of natural SARS-CoV-2 infections after the lifting of dynamic zero measures was not as strong as that of the neutralising antibodies produced after booster vaccination. This phenomenon may be due to the special character of COVID-19 and needs to be verified using larger sample sizes and more rigorous trials.

The humoral response observed within 690 days of the first booster vaccine dose supports the effectiveness of the heterologous manufacturer (type) approach (4). Although vaccine receipts in the heterologous-type vaccination group reported a higher incidence of adverse events, the side effects associated with the heterologous vaccination were within the range of those reported for homologous vaccination. The observed result of the solicited adverse events of the booster vaccine in this study was similar to that of a previous study (17). Neutralising antibody levels typically increase more after booster administration using a heterologous booster vaccine than that of homologous booster group in our study, which was similar with other mRNA booster heterologous vaccination (23–26). However, the mechanism of neutralizing antibody difference between two types of booster vaccination was unclear to need further explore.

A recent report from the United States Center for Disease Control and Prevention (CDC) revealed that the vaccine efficiency in people who had received the first booster dose of mRNA vaccines declined from 87% to 31% after 5 months in the omicron variant epidemic period (15). This result is similar to that of our study, indicating that recipients are increasingly susceptible to infection with SARS-CoV-2 with a decrease in neutralising antibody levels by 6–7 months post-booster vaccination.

Associations between blood type and neutralising antibody production or breakthrough infection rate among medical staff were not investigated in this study. However, previous studies have reported that patients with blood group A have an increased risk of SARS-CoV-2 infection, whereas those with blood group O have a decreased risk (27, 28). In the hospital inpatients in this study, the SARS-CoV-2-infection rate showed that since April 2024, the natural SARS-CoV-2-infection rate among the general population is no longer accurately following the previous cycle of one outbreak every 6 months, perhaps indicating that booster vaccinations break the link between blood types and infection susceptibility or involve COVID-19 outbreak cycles.

In the present study, we found that participants with an appropriate interval between vaccination doses (180–210 days) had elevated neutralising antibody levels than those with longer intervals (>210 days). This result supports the current 6–7-month booster interval for the COVID-19 vaccine, as vaccines produce fewer neutralising antibodies when the interval exceeds 7 months.

This study has some limitations. First, the number of participants was relatively small, and some participants had recall bias about whether they had experienced SARS-CoV-2 breakthrough infection, given that COVID-19 symptoms and signs can be confused with flu or the common cold in the absence of specific tests (29, 30). Additionally, in the population included in our study, fewer individuals received the heterologous type vaccine than those who received the homologous type vaccine, because when beginning the first booster vaccine, according to the booster vaccination guidelines released by the health administration, it was recommended that boosters receive the vaccine of the same manufacturer or homologous type as the primary immunization, in order to reduce the side effects of vaccination. Given our resource limitations, we could not conduct pseudo-virus neutralization tests for neutralising antibodies against the latest SARS-CoV-2 variant (31). Therefore, we could not determine the levels of neutralising antibodies against viruses causing breakthrough infections. We only found a trend for correlation between decreasing neutralising antibodies and an increasing rate of SARS-CoV-2 infection after vaccination over time. Some studies have shown that due to the emergence of the novel coronavirus variant KP.2,XEC, and XDV.1, the antibodies produced by the booster vaccine cannot neutralize the current variant strain (9–11). We speculate that the main reason is that the vaccine strain used in the booster vaccine is the prototype strain rather than the variant strain, which can also explain the high infection rate in the population despite the vaccination booster. In addition, the antibody and infection rate with SARS-CoV-2 for populations without vaccination for COVID-19 were not investigated; therefore, the comparison between the two population groups could not be calculated to obtain the protection rate of the booster vaccine. Moreover, we only investigated the antibody and breakthrough among medical staff in this study. Future studies should expand to more occupations, including workers or students, to compare the difference between antibody and breakthrough infection among different occupations. Furthermore, our results can be reported the neutralizing antibody response against to spike protein of SARS-CoV-2 to the currently Chinese COVID-19 booster vaccine used for this study, and not as a general message, perhaps a different antibody response against to nucleoprotein of SARS-CoV-2 (12, 13) was observed using a different vaccinate type, as mRNA vaccine (2, 14). We aim to include more participants and conduct pseudo-virus neutralization tests to address these limitations in the future.

In conclusion, this study evaluated the humoral immune responses and SARS-CoV-2 breakthrough infection within 690 days after the first booster vaccination dose in 389 medical staff members. Most participants rapidly developed increasing neutralising antibody levels after receiving a single second dose of the Chinese booster COVID-19 vaccine 1–2 weeks after the first. However, the neutralising antibody levels only last for 6–7 months following booster vaccination, after which the SARS-CoV-2-infection breakthrough rate increased significantly. We demonstrated relationships between blood type, age, sex, BMI, and duration after booster vaccination, reactivity, and breakthrough infection rate. Individuals with a longer antibody-level duration following booster vaccination and longer intervals between primary and booster vaccination after implementation of routine epidemic control measures had lower levels of antibodies and were more susceptible to infection with SARS-CoV-2 variants after the first booster dose than their counterparts. Our findings suggest that cooperation among researchers for the exploration of a broad spectrum and long duration neutralizing antibody of COVID-19 vaccines to booster population is a good idea to combat the immune escape of the increasing number of SARS-CoV-2 variants (32, 33).

## Data Availability

The original contributions presented in the study are included in the article/**Supplementary Material**, further inquiries can be directed to the corresponding authors.
