# Supplementary material for: Characteristics of humoral responses to the first coronavirus disease booster vaccine and breakthrough infection in central China: a multicentre, prospective, longitudinal cohort study
Source: Front Immunol. 2025 Jan 7;15:1446751. doi: 10.3389/fimmu.2024.1446751 (PMC11747216; doi:10.3389/fimmu.2024.1446751)
Supplement: Supplementary file 1 [file Table1.doc]

Supplementary Material

**Supplementary Material**

Supplementary Material Table 1. Factors associated with neutralizing antibody concentration following Chinese COVID-19 booster vaccination

| Factors | Days post-vaccination | | | | | | | | | | | |  | | | | |
| --- | --- | --- | --- | --- | --- | --- | --- | --- | --- | --- | --- | --- | --- | --- | --- | --- | --- |
|  | 1–14 (n = 99) | | | 15–30 (n = 81) | | | 31–90 (n = 129) | | | 91–150 (n = 155) | | |  | | | | |
|  | *n* | B (95%CI) | *p* | *n* | B (95%CI) | *p* | *n* | B (95%CI) | *p* | *n* | B (95%CI) | *p* |  | | | | |
| Sex | | | | | | | | | |  |  |  |  | | | | |
| Men | 40 | Reference |  | 11 | Reference |  | 28 | Reference |  | 51 | Reference |  |  | | | | |
| Women | 59 | 0.95  (0.5–1.5) | <0.001 | 70 | -0.1  (-0.2 to - 0.02) | 0.022 | 101 | -0.5  (-0.10 to 0.04) | 0.069 | 104 | -0.03  (-0.38 to 0.33) | 0.764 |  | | | | |
| Age (years) | | | | | | | | | |  |  |  |  | | |  | |
| 18–30 | 43 | Reference |  | 4 | Reference |  | 38 | Reference |  | 32 | Reference |  |  | | | | |
| 31–50 | 20 | 0.3  (-0.2 to 0.9) | 0.228 | 28 | -0.05  (-0.2 to 0.1) | 0.416 | 6 | 0.2  (-0.6 to 0.9) | 0.266 | 23 | 0.01  (-0.3 to 0.4) | 0.294 |  | | | | |
| > 50 | 36 | 0.05  (-0.5 to 0.6) | 0.865 | 49 | -0.03  (-0.2 to 0.1) | 0.646 | 85 | 0.3  (-1.1 to 0.5) | 0.241 | 100 | -0.11  (-0.39 to 0.8) | 0.283 |  | | | | |
| Blood type | | | | | | | | | |  |  |  |  | | |  |  |
| A | 39 | Reference |  | 13 | Reference |  | 59 | Reference |  | 61 | Reference |  |  | | | | |
| B | 16 | -0.6  (-1.1 to -0.2) | 0.003 | 6 | 0.1  (-0.04 to 0.2) | 0.291 | 24 | -0.4  (-0.7 to -0.1) | 0.043 | 21 | -0.37  (-0.8 to -0.1) | 0.063 |  | | | | |
| O | 6 | -0.2  (-0.8 to 0.5) | 0.663 | 34 | -0.05  (-0.1 to 0.05) | 0.516 | 34 | -0.3  (-1.3 to 0.6) | 0.170 | 12 | 0.3  (0.05 to 0.48) | 0.018 |  | | | | |
| AB | 38 | -0.06  (-0.8 to 0.6) | 0.873 | 28 | 0.01  (-0.1 to 0.1) | 0.611 | 12 | 0.3  (-0.8 to 1.3) | 0.703 | 61 | 0.2  (-0.33 to 0.72) | 0.057 |  | | | | |
| Vaccination type | | | | | | | | | | | | |  | | |  |  |
| Homologous type | 92 | Reference |  | 64 | Reference |  | 102 | Reference |  | 138 | Reference |  |  | | | | |
| Heterologous type | 7 | -0.1  (-0.6 to 1.5) | 0.884 | 17 | -0.13  (-0.2 to -0.01) | 0.001 | 27 | 0.77  (0.06 to 1.48) | 0.001 | 17 | 0.37  (0.1 to 0.58) | 0.001 |  | | | | |
| BMI (kg/m^2^) | | | | | | | | | | | | |  | | | | |
| < 18.5 | 0 | - |  | 0 | - |  |  | Reference |  | 0 | - |  |  | | | | |
| 18.5–23.9 | 64 | Reference |  | 59 | Reference |  | 97 | -0.3  (-1.3 to 0.7) | 0.546 | 97 | Reference |  |  | | | | |
| > 23.9 | 35 | 0.68  (0.3 to 1.11) | 0.001 | 22 | -0.1  (-0.2 to -0.02) | 0.018 | 29 | -0.3  (-0.8 to 0.3) | 0.288 | 58 | 0.16  (0.08 to 0.39) | 0.021 |  | | | | |
| Breakthrough infection | | | | | | | | | | | | |  |  |  | | |
| Yes | 15 | Reference |  | 4 | Reference |  | 10 | Reference |  | 19 | Reference |  |  | | | | |
| No | 84 | -0.02  (-0.8 to 0.7) | 0.902 | 77 | 0.1  (-0.06 to 0.16) | 0.354 | 119 | 0.38  (-0.5 to 1.3) | 0.154 | 136 | 0.06  (-0.6 to 0.7) | 0.057 |  | | | | |
| Dynamic zero policy | | | | | | | | | | | | |  | | | | |
| Before (Dec 13, 2022) | 84 | Reference |  | 77 | Reference |  | 115 | Reference |  | 136 | Reference |  |  | | | | |
| After (Dec13, 2022) | 15 | -0.02  (-0.3 to 0.3) | 0.902 | 4 | 0.1  (-0.06 to 0.16) | 0.354 | 14 | -0.8  (-1.3 to -0.4) | <0.001 | 19 | 0.06  (-0.6 to 0.7) | 0.057 |  | | | | |
| Interval between primary and booster dose of vaccination | | | | | | | | | | | | |  | | | | |
| 180–210 | 64 | Reference |  | 35 | Reference |  | 47 | Reference |  | 64 | Reference |  |  | | | | |
| >210 | 6 | 0.02  (-0.6 to 0.6) | 0.461 | 19 | 0.01  (-0.1 to 0.1) | 0.317 | 27 | -0.2  (-0.7 to 0.2) | 0.540 | 6 | -0.07  (-0.5 to 0.37) | 0.460 |  | | | | |

–, not available; BMI, body mass index

**Supplementary Material Table 1. Factors associated with neutralizing antibody concentration following Chinese COVID-19 booster vaccination (continued)**

| Factors | Days post-vaccination | | | | | | | | | | | |
| --- | --- | --- | --- | --- | --- | --- | --- | --- | --- | --- | --- | --- |
|  | 151–210 (*n* = 27) | | | 211–300 (*n* = 25) | | | 301–365 (*n* = 68) | | | 366–420 (n = 75) | | |
|  | *n* | B (95%CI) | *p* | *n* | B (95%CI) | *p* | *n* | B (95%CI) | *p* | *n* | B (95%CI) | *p* |
| Sex | | | | | | | | | |  |  |  |
| Men | 12 | Reference |  | 4 | Reference |  | 9 | Reference | 0.906 | 22 | Reference |  |
| Women | 15 | -0.4  (-3.2 to 2.3) | 0.770 | 21 | -2.9  (-7.0 to 1.1) | 0.151 | 59 | -0.1  (-1.3 to 1.1) |  | 53 | 0.6  (-0.5 to 1.7) | 0.66 |
| Age (years) | | | | | | | | | |  |  |  |
| 18–30 | 12 | Reference |  | 6 | Reference |  | 18 | Reference |  | 37 | Reference |  |
| 31–50 | 12 | 0.36  (-1.7 to 2.4) | 0.737 | 11 | 3.6  (0.1 to 7.0) | 0.041 | 38 | −0.62  (-1.2 to 0.10) | 0.249 | 17 | 0.5  (-0.5 to 1.5) | 0.711 |
| > 50 | 3 | -0.3  (-2.1 to 1.5) | 0.727 | 8 | 1.9  (-0.3 to 4.0) | 0.096 | 12 | -0.59  (-1.2 to 0.01) | 0.865 | 21 | 0.7  (-0.9 to 2.3) | 0.133 |
| Blood type | | | | | | | | | |  |  |  |
| A | 7 | Reference |  | 5 | Reference |  | 17 | Reference |  | 23 | Reference |  |
| B | 7 | 1.56  (-2.2 to 5.3) | 0.077 | 8 | 2.6  (-0.2 to 5.3) | 0.065 | 12 | 0.87  (-0.2 to 2.0) | 0.117 | 15 | 1.5  (0.2 to 2.9) | 0.042 |
| O | 8 | 0.19  (-0.3 to 0.66) | 0.059 | 11 | 0.5  (-1.0 to 2.0) | 0.493 | 35 | 1.0  (0.17 to 1.93) | 0.020 | 34 | 0.1  (-1.0 to 1.2) | 0.692 |
| AB | 5 | 0.3  (-0.33 to 0.72) | 0.669 | 1 | 3.3  (-0.3 to 6.9) | 0.075 | 4 | 0.11  (-1.1 to 0.9) | 0.976 | 3 | 1.0  (-1.2 to 3.3) | 0.648 |
| Vaccination type | | | | | | | | | |  |  |  |
| Homologous type | 14 | Reference |  | 15 | Reference |  | 41 | Reference | 0.075 | 46 | Reference |  |
| Heterologous type | 13 | -0.38  (-1.9 to 1.2) | 0.634 | 10 | 0.4  (-1.1 to 1.8) | 0.627 | 27 | 0.71  (-0.1 to 1.5) |  | 29 | 0.14  (-0.9 to 0.1) | 0.546 |
| BMI (kg/m^2^) |  | | | | | | | | | | | |
| < 18.5 |  | Reference |  | 5 | Reference |  | 4 | Reference |  | 6 | Reference |  |
| 18.5–23.9 | 16 | -2.8  (-6.2 to 0.8) | 0.128 | 18 | -3.3  (-5.0 to -1.6) | 0.001 | 33 | 2.8  (0.6 to 4.2) | 0.001 | 40 | -1.76  (-3.5 to -0.4) | 0.024 |
| > 23.9 | 11 | -1.6  (-0.34 to 0.2) | 0.091 | 2 | -7.9  (-13.3 to -2.5) | 0.004 | 31 | 3.3  (0.8 to 4.7) | 0.001 | 29 | -1.7  (-3.5 to 0.1) | 0.172 |
| Breakthrough |  |  |  |  |  |  |  |  |  |  |  |  |
| Yes | 3 | Reference |  | 22 | Reference |  | 58 | Reference |  | 56 | Reference |  |
| No | 24 | 1.1  (-1.7 to 3.8) | 0.461 | 3 | -0.1  (-2.0 to 1.76) | 0.896 | 10 | -0.36  (-2.2 to 1.53) | 0.284 | 19 | -0.24  (-1.3 to 0.8) | 0.696 |
| Dynamic zero policy | | | | | | | | | | | | |
| Before (Dec 13, 2022) | 3 | Reference |  | 2 | Reference |  | 4 | Reference |  | 4 | Reference |  |
| After (Dec 13, 2022) | 24 | 0.22  (-2.8 to 3.3) | 0.885 | 23 | -0.6  (-2.7 to 1.6) | 0.618 | 64 | -0.8  (-2.4 to 0.9) | 0.348 | 71 | 0.18  (-0.7 to 1.1) | 0.297 |
| Interval between primary and booster dose of vaccination | | | | | | | | | | | | |
| 180–210 | 17 | Reference |  | 15 | Reference |  | 42 | Reference |  | 56 | Reference |  |
| > 210 | 12 | 0.02  (-0.6 to 0.6) | 0.711 | 10 | -3.6  (-6.5 to -0.7) | 0.015 | 26 | -0.5  (-1.4 to 0.4) | 0.335 | 19 | 0.11  (-0.9 to 1.2) | 0.702 |

–, not available; BMI, body mass index

Supplementary Material Table 1. Factors associated with neutralizing antibody concentration following Chinese COVID-19 booster vaccination (continued)

| Factors | | Days post-vaccination | | | | | |
| --- | --- | --- | --- | --- | --- | --- | --- |
|  |  | 421–480 (*n* = 100) | | | 480-690 (*n* = 52) | | |
|  | | *n* | B (95%CI) | *p* | *n* | B (95%CI) | *p* |
| Sex |  | | | | | | |
| Men | | 42 | Reference |  | 11 | Reference |  |
| Women | | 58 | 0.1 (-0.7 to 0.9) | 0.867 | 41 | -0.2 (−0.78 to 0.33) | 0.612 |
| Age (years) | | | | | | | |
| 18–30 | | 42 | Reference |  | 34 | Reference |  |
| 31–50 | | 21 | 0.1 (-0.7 to 0.7) | 0.465 | 12 | 0.2 (−0.45 to 0.81) | 0.597 |
| > 50 | | 37 | −0.2 (-0.9 to 0.5) | 0.565 | 6 | -0.11 (−0.39 to 0.8) | 0.979 |
| Blood type | | | | | | | |
| A | | 16 | Reference |  | 12 | Reference |  |
| B | | 15 | -0.1 (-0.8 to 0.6) | 0.801 | 18 | 0.56 (0.1 to 1.2) | 0.031 |
| O | | 29 | -0.1 (-0.8 to 0.5) | 0.717 | 16 | 0.4(-0.2 to 0.88) | 0.068 |
| AB | | 40 | -0.5 (-1.2 to 0.3) | 0.208 | 6 | 0.5 (-0.16 to 0.3) | 0.188 |
| Vaccination type | | | | | | | |
| Homologous type | | 78 | Reference |  | 30 | Reference |  |
| Heterologous type | | 22 | -0.1 (-0.6 to 0.5) | 0.439 | 22 | 0.14 (-0.4 to 0.65) | 0.864 |
| BMI (kg/m^2^) | | | | | | | |
| <18.5 | | 6 | Reference |  | 3 | Reference |  |
| 18.5–23.9 | | 50 | -0.4 (-1.2 to 0.4) | 0.354 | 37 | -0.3 (-1.2 to 0.6) | 0.655 |
| > 23.9 | | 44 | -1.1 (-2.2 to -0.1) | 0.029 | 12 | -0.4 (-1.2 to 0.4) | 0.941 |
| Breakthrough | | | | | | | |
| Yes | | 72 | Reference |  | 47 | Reference |  |
| No | | 28 | 0.6 (-0.2 to 1.5) | 0.117 | 5 | -0.6 (-1.3 to 0.1) | 0.062 |
| Dynamic zero policy | | | | | | | |
| Before (Dec 13, 2022) | | 22 | Reference |  | 0 | Reference |  |
| After (Dec 13, 2022) | | 78 | 0.08 (-1.7 to 1.9) | 0.470 | 52 | - | - |
| Interval between primary and booster dose of vaccination | | | | | | | |
| 180–210 | | 66 | Reference |  | 23 | Reference |  |
| > 210 | | 34 | -1.1 (-1.6 to -0.6) | 0.001 | 29 | -0.4 (-0.6 to 0.1) | 0.343 |

–, not available; BMI, body mass index

Supplementary Material Table 2. Kinetics of neutralizing antibody concentration following booster vaccination according to blood type

| Factors |  | Days post-vaccination | | | | | | | | | | | |
| --- | --- | --- | --- | --- | --- | --- | --- | --- | --- | --- | --- | --- | --- |
|  | Before booster | 1–14 | 15–30 | 31–90 | 91–150 | 151–210 | 211–300 | 301–365 | 366–420 | 421–480 | 481-690 | F | *p* |
| Average | 13.3 ± 1.4 | 85.6 ± 1.1 | 98.4 ±1.1 | 96.6 ± 1.1 | 95.6 ± 1.1 | 95.6 ±1.3 | 37.6 ± 3.2 | 38.5 ± 1.3 | 75.8 ± 1.3 | 84.4 ±1.3 | 89.6 ±1.1 | 20.8 | <0.001 |
| A | 14.2 ± 1.4 | 88.3 ± 1.2 | 96.9 ± 1.3 | 93.8 ± 1.2 | 91.2 ± 1.2 | 93.1 ± 1.2 | 32.9 ±3.6 | 27.7 ± 1.5 | 57.3 ± 1.5 | 94.6 ±1.3 | 59.7 ±1.2 | 9.18 | <0.001 |
| B | 14.4 ± 1.3 | 60.4 ± 1.2 | 99.9 ± 1.3 | 64.9 ± 1.3 | 79.8 ± 1.2 | 93.4 ± 1.3 | 35.9 ±3.2 | 48.2 ± 1.5 | 98.9 ± 1.5 | 94.2 ±1.4 | 92.4 ±1.3 | 2.27 | 0.021 |
| O | 8.9 ± 1.4 | 88.3 ± 1.2 | 93.7 ± 1.2 | 73.5 ± 1.3 | 99.7 ± 1.1 | 99.9 ± 1.2 | 37.9 ±3.7 | 57.3 ± 1.4 | 49.5 ± 1.4 | 95.8 ±1.3 | 84.4 ±1.3 | 9.73 | <0.001 |
| AB | 17.1 ± 1.5 | 94.9 ± 1.2 | 97.1 ± 1.4 | 99.0 ± 1.4 | 99.3 ± 1.2 | 91.3 ± 1.2 | 35.6 ±3.9 | 28.2 ± 1.9 | 81.6±1.9 | 69.1 ±1.4 | 84.1±1.2 | 3.41 | <0.001 |
| LSMD  (95%CI) | - | 30.5  (11.0–35.2)^b^,  30.5  (3.5–33.9) ^c^ | - | - | - | - | - | 29.4  (10.1–36.3)^d^ | 41.6  (20.1–70.2)^e^,  24.3  (20.9–52.3) ^f^ | - | - |  |  |
| *F* | 1.287 | 1.464 | 0.612 | 1.076 | 2.278 | 0.252 | 0.228 | 1.945 | 2.809 | 2.694 | 1.116 |  |  |
| *p* | 0.623 | 0.014^b^, _0.037_^c^ | 0.059 | 0.682 | 0.006^e^, 0.029^f^ | 0.859 | 0.876 | 0.020^d^ | 0.006^e^, 0.042^f^ | 0.623 | 0.173 |  |  |

^a^Values were evaluated using a Heterologous line effect model adjusted for vaccine manufacturer, sex, age, and BMI. Log2 amount of the neutralizing antibody was used as the independent variable.

^b^Blood types B and A vs type A.

^c^Blood type AB vs type A

^d^ Blood type O vs type A

^e,f^ Blood types B and A vs type O

LSMD, least square mean difference; BMI, body mass index; -, not available.

Supplementary Material Table 3. Kinetics of neutralizing antibody concentratio^n^ following booster vaccination according to body mass index

| Factors |  | Days post-vaccination | | | | | | | | | | | |
| --- | --- | --- | --- | --- | --- | --- | --- | --- | --- | --- | --- | --- | --- |
|  | Before booster | 1–14 | 15–30 | 31–90 | 91–150 | 151–210 | 211–300 | 301–365 | 366–420 | 421–480 | 481–690 | *F* | *p* |
| Average | 13.3 ± 1.4 | 85.6 ± 1.1 | 98.4 ±1.1 | 96.6 ± 1.1 | 95.6 ± 1.1 | 95.6 ±1.3 | 37.6 ± 3.2 | 38.5 ± 1.3 | 75.8 ± 1.3 | 84.4 ± 1.3 | 89.6 ± 1.1 | 20.79 | <0.001 |
| <18.5 kg/m^2^ | 19.6 ± 1.3 | - | - | 78.2 ± 1.2 | - | 95.3 ± 1.1 | 40.5 ± 3.9 | 9.2 ± 1.8 | 98.7 ± 1.4 | 90.5 ± 1.3 | 83.9 ± 1.2 | 18.3 | <0.001 |
| 18.5–23.9 kg/m^2^ | 12.3 ± 1.3 | 99.1 ± 1.2 | 99.9 ± 1.3 | 99.7 ± 1.3 | 99.8 ± 1.1 | 96.5 ± 1.2 | 31.6 ± 3.8 | 48.5 ± 1.4 | 42.2 ± 1.5 | 90.6 ± 1.2 | 74.1 ± 1.3 | 22.4 | <0.001 |
| >24 kg/m^2^ | 9.6 ± 1.5 | 66.4 ± 1.1 | 99.5 ± 1.2 | 87.2 ± 1.3 | 90.5 ± 1.2 | 95.3 ± 1.3 | 15.3±3.1 | 61.8±1.4 | 69.5±1.3 | 57.4±1.3 | 81.1 ±1.1 | 5.42 | <0.001 |
| LSMD  (95%CI) | - | 32.7  (21.6,43.3) | - | - | 9.3 (6.1,12.2) | - | - | ^b^52.6  (22.3,68.4), ^c^39.3  (21.3,46.7) | - | ^b^40.2  (22.5,64.3),  ^c^49.1  (21.1,65.7) | - |  |  |
| F | 1.213 | 0.348 | 1.201 | 2.659 | 2.309 | 1.444 | 2.842 | 6.979 | 3.462 | 6.255 | 2.344 |  |  |
| p | 0.226 | 0.001 | 0.068 | 0.568 | 0.021 | 0.257 | 0.08 | 0.007^b^,0.01^c^ | 0.054 | 0.029^b^,0.009^c^ | 0.798 |  |  |

^a^Values were evaluated using a Heterologous-line effect model adjusted for vaccine manufacturer, sex, age, blood type, and occupation. Log2 amount of the neutralizing antibody was used as the independent variable.

LSMD, least square mean difference; -, not available.

**^b^**: >24 kg/m^2^VS <18.5 kg/m^2^

**^C^**:18.5–23.9 kg/m^2^VS <18.5 kg/m^2^

Supplementary Material Table 4. Kinetics of neutralizing antibody^a^ concentration following booster vaccination, according to vaccine types

| Factors | Before booster | Days post-vaccination | | | | | | | | | | | |
| --- | --- | --- | --- | --- | --- | --- | --- | --- | --- | --- | --- | --- | --- |
|  |  | 1–14 | 15–30 | 31–90 | 91–150 | 151–210 | 211–300 | 301–365 | 366–420 | 421–480 | 481–690 | F | *p* |
| Average | 13.3 ± 1.4 | 85.6 ± 1.1 | 98.4 ± 1.1 | 96.6 ± 1.1 | 95.6 ± 1.1 | 95.6 ± 1.3 | 37.6 ± 3.2 | 38.5 ± 1.3 | 75.8 ± 1.3 | 84.4 ± 1.3 | 89.6 ± 1.1 | 20.79 | <0.001 |
| Homologous type | 20.5 ± 1.1 | 83.0 ± 3.1 | 95.7 ± 2.5 | 64.9 ± 2.5 | 90.2 ± 1.2 | 70.7 ± 1.2 | 29.9 ± 3.3 | 30.7 ± 1.3 | 68.4 ± 1.4 | 78.8 ± 1.4 | 78.7 ± 1.2 | 18.6 | <0.001 |
| Heterologous type | 8.5 ± 1.7 | 86.1 ± 3.0 | 99.7 ± 2.9 | 97.8 ± 2.2 | 95.9 ± 1.1 | 97.2 ± 1.2 | 43.9 ± 3.2 | 48.2 ± 1.4 | 83.9 ± 1.5 | 90.5 ± 1.3 | 81.0 ± 1.3 | 5.14 | <0.001 |
| LSMD (95%CI) | 12.0  (7.2–19.5) | - | - | 32.9  (22.1–43.1) | 5.7  (3.6–10.7) | - | - | - | - | - | - |  |  |
| t | 2.403 | 0.880 | 0.142 | 2.363 | 2.18 | 0.824 | 0.792 | 1.860 | 0.628 | 1.228 | 1.128 |  |  |
| p | 0.212 | 0.884 | 0.58 | <0.001 | 0.001 | 0.418 | 0.436 | 0.075 | 0.786 | 0.867 | 0.864 |  |  |

^a^Values were calculated and based on a Heterologous line effect model adjusted for age, sex, blood type, occupation, and body mass index. Log2 amount of neutralizing antibody was the independent variable.

LSMD, least squares mean difference. -, not available.

Supplementary Material Table 5. Kinetics of neutralization antibody concentration^a^ following booster vaccination according to sex

| Factors |  | Days post-vaccination | | | | | | | | | | | |
| --- | --- | --- | --- | --- | --- | --- | --- | --- | --- | --- | --- | --- | --- |
|  | Before booster | 1–14 | 15–30 | 31–90 | 91–150 | 151–210 | 211–300 | 301–365 | 366–420 | 421–480 | 481–690 | F | p |
| Average | 13.3 ±1.4 | 85.6 ± 1.1 | 98.4 ±1.1 | 96.6 ± 1.1 | 95.6 ± 1.1 | 95.6 ±1.3 | 37.6 ± 3.2 | 38.5 ± 1.3 | 75.8 ± 1.3 | 84.4 ±1.3 | 89.6 ±1.1 | 20.79 | <0.001 |
| Men | 10.4 ±1.4 | 60.8 ± 1.2 | 97.8 ± 1.3 | 89.3 ± 1.3 | 64.9 ± 1.2 | 91.4 ± 1.3 | 37.1± 3.1 | 37.3±1.5 | 70.0±1.5 | 82.8 ±1.3 | 79.9 ±1.3 | 4.913 | <0.001 |
| Women | 16.9 ±1.3 | 91.1 ± 1.1 | 99.9 ± 1.2 | 97.5 ± 1.2 | 97.3 ± 1.1 | 97.1 ± 1.1 | 39.9 ± 3.4 | 39.5 ±1.1 | 81.6±1.4 | 84.7 ±1.3 | 85.9 ± 1.2 | 16.33 | <0.001 |
| LSMD  (95%CI) | 6.5  (5.1–10.1) | 30.3  (21.8–54) | - | - | 32.4  (22.3–45.1) | - |  | - | - | - | - | - | - |
| t | 2.212 | 1.143 | 0.618 | 0.726 | 1.185 | 0.289 | 0.071 | 1.656 | 1.143 | 1.732 | 1.343 | - | - |
| p | <0.001 | <0.001 | 0.967 | 0.069 | 0.001 | 0.775 | 0.944 | 0.916 | 0.294 | 0.783 | 0.612 | - | - |

^a^Values were calculated based on a Heterologous-line effect model adjusted for vaccine manufacturers, age, blood type, and BMI. Log2 amount of the neutralizing antibody was used as the independent variable.

LSMD, least squares mean difference; -, not available.

Supplementary Material Table 6. Kinetics of neutralizing antibody concentration^a^ following booster vaccination according to age

| Factors |  | Days post-vaccination | | | | | | | | | | | |
| --- | --- | --- | --- | --- | --- | --- | --- | --- | --- | --- | --- | --- | --- |
|  | Before booster | 1–14 | 15–30 | 31–90 | 91–150 | 151–210 | 211-300 | 301–365 | 366–420 | 421–480 | 481-690 | F | p |
| Average age | 13.3 ±1.4 | 85.6 ± 1.1 | 98.4 ±1.1 | 96.6 ± 1.1 | 95.6 ± 1.1 | 95.6 ±1.3 | 37.6 ± 3.2 | 38.5 ± 1.3 | 75.8 ± 1.3 | 84.4 ± 1.3 | 89.6 ± 1.1 | 20.79 | <0.001 |
| 18–30 years | 19.3 ±1.3 | 75.3 ± 1.3 | 99.1 ± 1.3 | 98.8 ± 1.2 | 95.1 ± 1.1 | 61.5 ± 1.3 | 62.8 ± 3.8 | 42.2 ± 1.4 | 59.7 ± 1.4 | 82.8 ± 1.2 | 82.1 ± 1.2 | 1.799 | <0.001 |
| 31–50 years | 12.3 ±1.3 | 88.1 ± 1.2 | 99.7 ± 1.3 | 87.2 ± 1.3 | 99.2 ± 1.2 | 99.1 ± 1.2 | 25.9 ± 3.6 | 29.3 ± 1.4 | 69.6 ± 1.7 | 74.7 ± 1.2 | 74.5 ± 1.3 | 4.543 | <0.001 |
| >50 years | 11.3 ±1.5 | 82.4 ± 1.1 | 99.7 ± 1.2 | 95.3 ± 1.3 | 89.2 ± 1.3 | 78.7 ± 1.2 | 42.8 ± 3.1 | 45.2 ± 1.3 | 85.5± 1.6 | 94.6 ± 1.3 | 82.7 ± 1.3 | 2.504 | <0.001 |
| LSMD (95%CI) | 8.0  (7.1–12.5) | - | - | - | - | - |  | - | - | - | - | - | - |
| F | 2.710 | 0.432 |  | 2.649 | 0.732 | 0.431 | 1.179 | 1.695 | 1.246 | 0.316 | 0.366 | - | - |
| P | 0.01 | 0.356 |  | 0.127 | 0.120 | 0.655 | 0.326 | 0.193 | 0.295 | 0.833 | 0.857 | - | - |

^a^Values were calculated based on a Heterologous-line effect model adjusted for vaccine manufacturers, sex, BMI, and blood type. Log2 amount of the neutralizing antibody was used as the independent variable.

^b^Age: >50 years vs 18–30 years.

^c^Age: 31-50 years vs 18–30 years.

LSMD, least square mean difference; -, not available.

Supplementary Material Table 7. Kinetics of neutralizing antibody concentration^a^ following booster vaccination according to breakthrough infection with SARS-CoV-2

| Factors |  | Days post-vaccination | | | | | | | | | | | |
| --- | --- | --- | --- | --- | --- | --- | --- | --- | --- | --- | --- | --- | --- |
|  | Before booster | 1–14 | 15–30 | 31–90 | 91–150 | 151–210 | 211-300 | 301–365 | 366–420 | 421–480 | 481-690 | F | p |
| Average | 13.3 ± 1.4 | 85.6 ± 1.1 | 98.4 ± 1.1 | 96.6 ± 1.1 | 95.6 ± 1.1 | 95.6 ± 1.3 | 37.6 ± 3.2 | 38.5 ± 1.3 | 75.8 ± 1.3 | 84.4 ± 1.3 | 89.6 ± 1.1 | 20.79 | <0.001 |
| Infection | - | 94.0 ± 1.3 | 99.9 ± 1.3 | 90.1 ± 1.2 | 89.9 ± 1.1 | 61.5 ± 1.3 | 35.0 ± 3.1 | 30.7 ± 1.4 | 81.1 ± 1.4 | 74.5 ± 1.2 | 95.7 ± 1.3 | 5.59 | <0.001 |
| Non-infection | 13.3 ± 1.4 | 82.3 ± 1.2 | 79.7 ± 1.3 | 98.0 ± 1.3 | 99.2 ± 1.2 | 99.1 ± 1.2 | 62.2 ± 3.3 | 48.2 ± 1.4 | 71.1 ± 1.7 | 95.7 ± 1.2 | 70.3 ± 1.2 | 11.84 | <0.001 |
| LSMD (95%CI) | - | - | - | - | - | - | - | - | - | - | - |  |  |
| T | - | 0.432 | 1.103 | 1.649 | 0.732 | 0.431 | 1.062 | 1.695 | 1.246 | 0.316 | 0.567 |  |  |
| P | - | 0.902 | 0.354 | 0.154 | 0.057 | 0.655 | 0.358 | 0.193 | 0.295 | 0.833 | 0.062 |  |  |

^a^Values were calculated based on a Heterologous-line effect model adjusted for vaccine manufacturers, sex, body mass index, and blood type. Log2 amount of the neutralizing antibody was used as the independent variable.

^b^Age: >50 years vs 18-30 years.

^c^Age: 31-50 years vs 18–30 years.

LSMD, least square mean difference; -, not available.

Supplementary Material Table 8. Kinetics of neutralizing antibody concentration^a^ following booster vaccination according to vaccination interval

| Factors |  | Days post-vaccination | | | | | | | | | | | |
| --- | --- | --- | --- | --- | --- | --- | --- | --- | --- | --- | --- | --- | --- |
|  | Before booster | 1–14 | 15–30 | 31–90 | 91–150 | 151–210 | 211–300 | 301–365 | 366–420 | 421–480 | 481–690 | F | p |
| Average | 13.3±1.4 | 85.6 ± 1.1 | 98.4 ± 1.1 | 96.6 ± 1.1 | 95.6 ± 1.1 | 95.6 ± 1.3 | 37.6 ± 3.2 | 38.5 ± 1.3 | 75.8 ± 1.3 | 84.4 ± 1.3 | 89.6 ± 1.1 | 20.79 | <0.001 |
| 180–210 | - | 95.1 ± 1.3 | 99.3 ± 1.3 | 98.6 ± 1.2 | 99.0 ± 1.1 | 96.1 ± 1.3 | 62.3 ± 3.7 | 44.0 ± 1.4 | 81.1± 1.4 | 90.6 ± 1.2 | 84.4 ± 1.2 | 26.98 | <0.001 |
| >210 | - | 78.2 ± 1.2 | 89.7 ± 1.3 | 94.6 ± 1.3 | 90.6 ± 1.2 | 91.5 ± 1.2 | 32.0 ± 3.2 | 33.6 ± 1.4 | 70.1 ± 1.7 | 57.7 ± 1.2 | 74.5 ± 1.3 | 3.82 | <0.001 |
| LSMD (95%CI) | - | - | - | - | - | - | - | - | - | 32.9 ^b^  (11.3–53.3) | - |  |  |
| T | - | 0.432 | 1.112 | 1.649 | 0.732 | 0.431 | 1.232 | 1.695 | 1.246 | 3.16 | 1.693 |  |  |
| p* |  | 0.461 | 0.317 | 0.54 | 0.460 | 0.655 | 0.231 | 0.193 | 0.295 | 0.01^a^ | 0.343 |  |  |

^a^Values were calculated based on a Heterologous-line effect model adjusted for vaccine manufacturers, sex, body mass index, and blood type. Log2 amount of the neutralizing antibody was used as the independent variable.

^b^interval:180-210 vs >210 days.

LSMD, least square mean difference; -, not available.

Table S9. Kinetics of neutralizing antibody concentration^a^ following booster vaccination according to epidemic policy

| Factors |  | Days post-vaccination | | | | | | | | | | | |
| --- | --- | --- | --- | --- | --- | --- | --- | --- | --- | --- | --- | --- | --- |
|  | Before booster | 1–14 | 15–30 | 31–90 | 91–150 | 151–210 | 211-300 | 301–365 | 366–420 | 421–480 | 481-690 | F | p |
| Average* | 13.3 ± 1.4 | 85.6 ± 1.1 | 98.4 ± 1.1 | 96.6 ± 1.1 | 95.6 ± 1.1 | 95.6 ± 1.3 | 37.6 ± 3.2 | 38.5 ± 1.3 | 75.8 ± 1.3 | 84.4 ±1.3 | 89.6 ±1.1 | 20.79 | <0.001 |
| Before Dec 13, 2022 | 13.3 ± 1.4 | 86.8 ± 1.3 | 92.1 ± 1.3 | 99.5 ± 1.2 | 99.6 ± 1.1 | 61.5 ± 1.3 | 35.0 ± 3.4 | 50.6 ± 1.4 | 96.7 ± 1.4 | 90.6 ± 1.2 | - | 1.84 | <0.001 |
| After Dec 13, 2022 | - | 81.3 ± 1.2 | 99.7 ± 1.3 | 82.2 ± 1.3 | 89.9 ± 1.2 | 96.4 ± 1.2 | 65.0 ± 3.2 | 29.2 ± 1.4 | 52.3 ± 1.7 | 78.7 ± 1.2 | 89.6 ± 1.1 | 6.32 | <0.001 |
| LSMD (95%CI) |  | - | 7.6  (3.1–11.9) | 17.3  (11.1–43.3) | - | - | 64.7  (16.6–78.9) | - | - | - |  |  |  |
| t |  | 0.432 | 3.103 | 2.649 | 0.732 | 0.431 | 4.248 | 1.695 | 1.246 | 0.316 | - |  |  |
| p |  | 0.461 | 0.012^b^ | 0.001^b^ | 0.057 | 0.655 | 0.001^b^ | 0.193 | 0.297 | 0.833 | - |  |  |

^a^Values were calculated based on a Heterologous-line effect model adjusted for vaccine manufacturers, sex, body mass index, and blood type. Log2 amount of the neutralizing antibody was used as the independent variable.

^b^ Before Dec,13,2022 vs after Dec,13, 2022.

LSMD, least square mean difference; -, not available.

Supplementary Material Table 10. Positivity rate of SARS-CoV-2 infection in hospitalized patients and other populations

| Date | Nov  2022 | Dec  1–12, 2022 | Dec 13–30, 2022 | Jan  2023 | Feb 2023 | Mar 2023 | Apr 2023 | May 2023 | Jun 2023 | Jul 2023 | Aug  2023 | Sep  2023 | Oct 2023 | Nov 2023 | Dec  2023 | Jan  2024 | Feb  2024 | Mar  2024 | April 2024 | May  2024 |
| --- | --- | --- | --- | --- | --- | --- | --- | --- | --- | --- | --- | --- | --- | --- | --- | --- | --- | --- | --- | --- |
| Number of detected people | 334103 | 97115 | 7427 | 12890 | 1809 | 998 | 743 | 2972 | 1942 | 972 | 699 | 861 | 583 | 750 | 1408 | 2180 | 320 | 1117 | 287 | 213 |
| Positive number of SARS-CoV-2 | 91 | 2023 | 3236 | 1218 | 98 | 34 | 190 | 1413 | 698 | 200 | 171 | 186 | 85 | 72 | 79 | 59 | 56 | 169 | 46 | 36 |
| Positive rate of SARS-CoV-2 infection | 0.27 | 2.83 | 43.57 | 10.1 | 5.4 | 3.4 | 25.6 | 47.5 | 35.9 | 20.6 | 24.5 | 21.6 | 14.6 | 9.6 | 5.6 | 2.7 | 17.5 | 15.1 | 16.0 | 16.9 |
